# Virtual Sensing of Key Variables in the Hydrogen Production Process: A Comparative Study of Data-Driven Models

**DOI:** 10.3390/s24103143

**Published:** 2024-05-15

**Authors:** Yating Yao, Yupeng Xing, Ziteng Zuo, Chihang Wei, Weiming Shao

**Affiliations:** 1 Department of Chemical Equipment and Control Engineering, College of New Energy, China University of Petroleum (East China), Qingdao 266580, China; s21150032@s.upc.edu.cn (Y.Y.); b22150006@s.upc.edu.cn (Y.X.); 2015010229@s.upc.edu.cn (Z.Z.); 2 School of Information Science and Technology, Hangzhou Normal University, Hangzhou 311121, China; weichihang@hznu.edu.cn

**Keywords:** hydrogen production process, data-driven virtual sensor, real-time estimation, variational Bayesian principal component analysis

## Abstract

Hydrogen is an ideal energy carrier manufactured mainly by the natural gas steam reforming hydrogen production process. The concentrations of ${\mathrm{CH}}_{4}$, CO, ${\mathrm{CO}}_{2}$, and $H_{2}$ in this process are key variables related to product quality, which thus need to be controlled accurately in real-time. However, conventional measurement methods for these concentrations suffer from significant delays or huge acquisition and upkeep costs. Virtual sensors effectively compensate for these shortcomings. Unfortunately, previously developed virtual sensors have not fully considered the complex characteristics of the hydrogen production process. Therefore, a virtual sensor model, called “moving window-based dynamic variational Bayesian principal component analysis (MW-DVBPCA)” is developed for key gas concentration estimation. The MW-DVBPCA considers complicated characteristics of the hydrogen production process, involving dynamics, time variations, and transportation delays. Specifically, the dynamics are modeled by the finite impulse response paradigm, the transportation delays are automatically determined using the differential evolution algorithm, and the time variations are captured by the moving window method. Moreover, a comparative study of data-driven virtual sensors is carried out, which is sporadically discussed in the literature. Meanwhile, the performance of the developed MW-DVBPCA is verified by the real-life natural gas steam reforming hydrogen production process.

## 1. Introduction

Hydrogen is an ideal energy storage medium with zero carbon emissions, essential in energy systems’ low-carbon transformation [1]. Currently, over 90% of the world’s large-scale industrial hydrogen production process is the fossil energy reforming process, of which more than 50% is the natural gas steam reforming process [2,3]. The natural gas steam reforming hydrogen production process consists of feedstock purification, steam reforming, medium temperature conversion, and pressure swing adsorption. In this process, ${\mathrm{CH}}_{4}$, CO, ${\mathrm{CO}}_{2}$, and $H_{2}$ are primary process gases, and the concentrations of these gases are key variables (KVs) related to the product quality. Infrequent and inaccurate measurements of these KVs risk substantial production losses, poor control performance, and even hidden dangers to safety. Consequently, real-time measurements of these KVs are essential and desirable.

Currently, measurements of the hydrogen production process’ KVs are classified into offline laboratory analyses, hardware sensors, and virtual sensors. The offline laboratory analyses give accurate measurements of the KVs, but result in significant measurement delays [4]. The hardware sensors measure the KVs in real-time, but need colossal investment and maintenance costs [5,6]. Virtual sensors are actually predictive mathematical models which use explanatory variables (EVs, i.e., easily measurable variables like pressure and flow rate) as inputs and estimates of the KVs as outputs, having the benefits of no measurement delays and low costs [7,8]. Therefore, the virtual sensor effectively compensates for the shortcomings of the offline laboratory analysis and hardware sensor [9,10]. As a result, virtual sensors have been intensively studied and widely used in the hydrogen production process.

Virtual sensors can generally be grouped into first-principle-based and data-driven virtual sensors. The first-principle-based virtual sensor is established by analyzing and explaining the physicochemical mechanisms of the hydrogen production process. For example, Yang et al. established a predictive model of ammonia pyrolysis decomposition rate through theoretical derivation for the reaction in the hydrogen production process [11]. Huang et al. developed a mathematical model between the EVs and the conversion rate of methane by coupling conduction, convection, thermal radiation, and chemical reaction kinetics [12]. Zhou et al. simulated the chemical looping hydrogen production process by analyzing the process mechanisms [13]. However, due to the complex reaction dynamics, precise first-principle-based models of the hydrogen production process are challenging to obtain. Usually, laboratory-scale studies or ideal steady-state analyses are the main applications of these first-principle-based virtual sensors. On the contrary, a data-driven virtual sensor is constructed by process-measured data, and does not rely on accurate mechanisms [14,15]. Therefore, it is closer to reality, and better describes the actual operations of the hydrogen production process [16,17]. As a result, data-driven virtual sensors are widely applied in the hydrogen production process. For example, Tong et al. built the multi-layer perceptron (MLP) neural network to estimate the ${\mathrm{CH}}_{4}$ concentration in the hydrogen production process [18]. Zamaniyan et al. employed the MLP model to predict the concentration of $H_{2}$ and CO of the hydrogen production process [19]. Ögren et al. established deep neural networks (DNNs) to estimate the concentration of ${\mathrm{CH}}_{4}$, CO, ${\mathrm{CO}}_{2}$, and $H_{2}$, which have large network layers [20]. Considering the challenges of artificial neural network models in model selection and parameter adjustment with limited data, small-data-oriented models have been applied to the hydrogen production process. Fang et al. used a support vector machine (SVM) to predict the hydrogen yield [21]. Zhao et al. proposed a predictive model based on the least squares SVM (LSSVM) employed in wind power hydrogen production [22].

Despite these achievements on virtual sensors for the KVs, the hydrogen production process is usually complicated because of practical operations. Firstly, due to the feedstock variations and external disturbances, the hydrogen production process shows strong dynamics [23]. Secondly, the hydrogen production process involves many series-wound devices (e.g., pre-reformers and pre-heaters), which requires significant time to deliver the energies and feedstocks to the reformer [24]. That means the EVs and the KVs are recorded simultaneously and are mismatched (i.e., there are transportation delays between the EVs and the KVs). Thirdly, the hydrogen production process exhibits time-varying properties because of the process drifts caused by mechanical abrasions, catalyst deactivation, or even climatic variations [25].

The above complex properties of the hydrogen production process make it challenging to develop high-precision virtual sensors for the KVs. As far as we know, the existing virtual sensors developed for the hydrogen production process do not account for these complicated characteristics. Moreover, the hydrogen production process has multiple KVs to estimate, and the existing virtual sensors constructed multiple independent single-output virtual sensors (one for each KV), ignoring the correlations between the KVs. To this end, we first develop a multi-output virtual sensor model called “dynamic variational Bayesian principal component analysis (DVBPCA)” for real-time prediction of the KVs in the hydrogen production process. As a further contribution, a moving window-based DVBPCA (MW-DVBPCA) is developed to improve estimation performance while considering the time variations of the hydrogen production process. The main contributions we make in the article are organized as follows.

(1) The developed DVBPCA considers both process dynamics and transportation delays of energies and materials. Concretely, the finite impulse response (FIR) method is employed to model the dynamics of the hydrogen production process. And the transportation delays related to the EVs are automatically determined by differential evolution (DE). Moreover, the DVBPCA is able to make full use of the correlations between KVs for performance enhancement.
(2) The moving window (MW) approach is employed for updating the DVBPCA with the latest online process information, which effectively captures the time-varying characteristics of the hydrogen production process in real-time.
(3) A comparative study of data-driven virtual sensors is implemented for the hydrogen production process, which is sparsely mentioned in the predecessors’ research. Furthermore, the performance of the developed MW-DVBPCA is verified by the real-life natural gas steam reforming hydrogen production process.

This article is structured as follows. The variational inference (VI) is introduced in Section 2. Section 3 describes the developed DVBPCA and MW-DVBPCA in detail. In Section 4, the performance of the DVBPCA and MW-DVBPCA is evaluated using the operational data from the distributed control system (DCS) database of a real-life hydrogen production process. Finally, Section 5 concludes the article.

## 2. Variational Inference

The VI and Monte Carlo–Markov Chain are common methods to study posterior distributions over RVs in probabilistic models [26]. And the VI was used to learn the developed DVBPCA, considering its advantages in convergence diagnosis and computational efficiency.

Denote the random variables (RVs) with unknown posterior distribution as Θ. The equation shown as Equation (1) decomposes the log of model evidence, as follows:

(1) $$\begin{array}{cc} \; & \ln p\left(D\right)=L\left(q\right)+\mathrm{KL}\left(q\|p\right)\\ \; & \mathrm{KL}\left(q\|p\right)=\int q\left(\Theta \right)\ln\left\{\frac{q\left(\Theta \right)}{p\left(\Theta |D\right)}\right\}d\Theta \\ \; & L\left(q\right)=\int q\left(\Theta \right)\ln\left\{\frac{p\left(D,\Theta \right)}{q\left(\Theta \right)}\right\}d\Theta \end{array}$$

where $\mathrm{KL}\left(q\|p\right)$ means the Kullback–Leibler (KL) divergence between $q\left(\Theta \right)$ and $p\left(\Theta |D\right)$, D is training dataset, $q\left(\Theta \right)$ represents any form of probability distribution over Θ, $p\left(\Theta |D\right)$ represents the actual posterior distribution over Θ, and $L\left(q\right)$ means the variational evidence lower bound (ELBO) of the log of model evidence $\ln P\left(D\right)$ as $\mathrm{KL}\left(q\|p\right)\geq 0$.

Therefore, the posterior distributions over Θ can be found by maximizing the ELBO $L\left(q\right)$ as shown in Equation (2), i.e.,

(2) $$p\left(\Theta |D\right)=q^{*}\left(\Theta \right)=\underset{q(\Theta )}{\arg\max}\;L\left(q\left(\Theta \right)\right)$$

where $q^{*}\left(\Theta \right)$ represents the variational solution of q(Θ).

Assume q(Θ) can factorize into the product $\prod_{j=1}^{J}q_{j}\left(\theta_{j}\right)$, where $\Theta =\{\theta_{1},\theta_{2},\cdots ,\theta_{J}\}$. The general rule to find the variational distribution $q_{j}\left(\theta_{j}\right)$ is obtained as Equation (3):

(3) $$\ln q_{j}^{*}\left(\theta_{j}\right)=\int \ln p\left(D,\Theta \right)\prod_{i\neq j}q_{i}^{*}\left(\theta_{i}\right)d\theta_{i}+C=E_{q_{i}\left(\theta_{i\neq j}\right)}\left[\ln p\left(D,\Theta \right)\right]+C$$

where $E_{q(\theta )}\left[f\left(\theta \right)\right]$ is the expectation of a function f(θ) of the RV θ with respect to the distribution q(θ) over θ, $\theta_{i\neq j}$ means factors in Θ except $\theta_{j}$, and C means constant terms.

## 3. Dynamic Variational Bayesian Principal Component Analysis Based on Moving Window

### 3.1. Time-Delayed Moving Average Model

Due to the flexibility of combining various regression models, the FIR paradigm is generally employed for capturing dynamics. Moreover, the KVs are measured at a lower frequency than the EVs in the hydrogen production process, which implies that the model structure based on autoregression from the FIR family is unsuitable. More importantly, as mentioned earlier, the transportation delays between the EVs and the KVs are unknown, and require consideration. Based on these considerations, a time-delayed moving average (MA) model structure is embedded in the virtual sensor, which is given by

(4) $$y\left(n\right)=f\left[\begin{array}{c} x_{1}\left(n-l_{1}\right),x_{1}\left(n-l_{1}-1\right),\cdots ,x_{1}\left(n-l_{1}-p_{1}\right),\\ x_{2}\left(n-l_{2}\right),x_{2}\left(n-l_{2}-1\right),\cdots ,x_{2}\left(n-l_{2}-p_{2}\right),\\ \vdots \\ x_{S}\left(n-l_{S}\right),x_{S}\left(n-l_{S}-1\right),\cdots ,x_{S}\left(n-l_{S}-p_{S}\right) \end{array}\right]$$

where $y\left(n\right)\in R^{K_{y}}$ are the *n*-th measurements of the $K_{y}$-dimensional KVs, $x_{i}\left(n\right)$ is *n*-th measurement of the *i*-th EV, *S* is the number of the EVs, $l_{i}$ and $p_{i}$ refer to the transportation delay and the order of $x_{i}$, respectively, and both are non-negative integers, and f(·) means the mathematical relationship between the EVs and KVs (but there may exist strong colinearities between the EVs of the model f(·)). Denote $l=(l_{1},l_{2},\cdots ,l_{S})$ and $p=(p_{1},p_{2},\cdots ,p_{S})$ for conciseness.

### 3.2. Dynamic Variational Bayesian Principal Component Analysis

Variational Bayesian principal component analysis (VBPCA) is a widely used model to deal with the colinearities between the EVs, owing to its advantage in the automatic determination of appropriate model dimensionality [27]. Therefore, it is ideally suited as the model f(·). In this article, name the VBPCA combined with the model structure shown in Equation (4) as the dynamic VBPCA (DVBPCA) due to the capability of capturing process dynamics. Moreover, the DVBPCA can handle the overfitting resulting from highly dimensional variable augmentation in Equation (4) through penalizing parameter values, which are detailed as follows.

**Figure 1:**
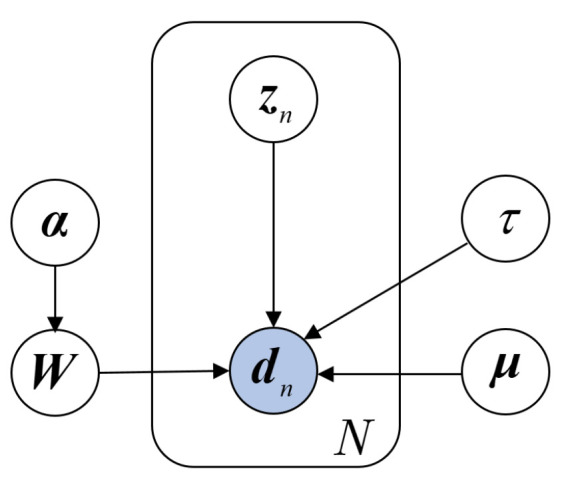
Probability graph of the DVBPCA.

Denote the observed sequential dataset as $D={\left(d_{1},d_{2},\cdots ,d_{N}\right)}^{T}$, where $d_{n}=\left(\begin{array}{c} x_{n}\\ y_{n} \end{array}\right)\in R^{K}$ means the *n*-th set of samples, $x_{n}={\left(v_{1}^{l_{1},p_{1}}\left(n\right),v_{2}^{l_{2},p_{2}}\left(n\right)\cdots ,v_{K_{x}}^{l_{K_{x}},p_{K_{x}}}\left(n\right)\right)}^{T}\in R^{K_{k}}$ and $y_{n}=y\left(n\right)\in R^{K_{y}}$ are inputs and outputs, respectively, and $v_{i}^{l_{i},p_{i}}\left(n\right)=\left(x_{i}\left(n-l_{i}\right),x_{i}\left(n-l_{i}-1\right),\cdots ,x_{i}\left(n-l_{i}-p_{i}\right)\right)$. Figure 1 graphically represents the DVBPCA. where, $W_{x}\in R^{K_{x}\times M}$ is the loading matrix of the inputs $x_{n}$, $W_{y}\in R^{K_{y}\times M}$ is the loading matrix of the outputs $y_{n}$, $W=\left(\begin{array}{c} W_{x}\\ W_{y} \end{array}\right)$, $z_{n}\in R^{M}$ are the *M*-dimensional hidden variables corresponding to the sample at time instant *n*, $\mu_{x}\in R^{K_{x}}$ are the mean values of the inputs $x_{n}$, $\mu_{y}\in R^{K_{y}}$ are the mean values of the outputs $y_{n}$, $\mu =\left(\begin{array}{c} \mu_{x}\\ \mu_{y} \end{array}\right)$, τ is the accuracy parameter of the noise, and $\alpha ={\left(\alpha_{1},\alpha_{2},\cdots ,\alpha_{M}\right)}^{T}$ is the accuracy parameter set of the loading matrix. Moreover, denote each column of W as $w_{m}$, the transpose of each row of W as $w_{k}^{*}$, and $Z={\left(z_{1},z_{2},\cdots ,z_{N}\right)}^{T}$. As illustrated in Equation (5), the observed data point $d_{n}$ is assumed to be generated by

(5) $$d_{n}=Wz_{n}+\mu +\varepsilon_{n}$$

where $\varepsilon_{n}$ is the measurement noise.

As shown in Equation (6), assign conjugate prior to the hidden variables

(6) $$p\left(z_{n}\right)=N\left(z_{n}|0_{M},I_{M}\right)$$

where N(·) represents the normal distribution probability density function, $0_{M}$ represents an *M*-dimensional column vector with element 0, and $I_{M}$ represents an *M*-dimensional unit matrix.

According to Figure 1, which shows that the samples are independently collected, the conditional distributions of the observed samples are designed as shown in Equations (7); that is,

(7) $$p\left(D|Z,W,\mu ,\tau \right)=\prod_{n=1}^{N}N\left(d_{n}|Wz_{n}+\mu ,\tau^{-1}I_{K}\right)$$

where $I_{K}$ represents a *K*-dimensional unit matrix.

To conform with common sense and to simplify subsequent calculations, the distributions over all RVs are selected as conjugate priors as detailed in Equation (8)–(11); that is,

(8) $$p\left(W|\alpha \right)=\prod_{m=1}^{M}N\left(w_{m}|0_{K},\alpha_{m}^{-1}I_{K}\right)$$

(9) $$p\left(\mu \right)=N\left(\mu |0_{K},\beta^{-1}I_{K}\right)$$

(10) $$p\left(\alpha \right)=\prod_{m=1}^{M}G\left(\alpha_{m}|a_{\alpha },b_{\alpha }\right)$$

(11) $$p\left(\tau \right)=G\left(\tau |a_{\tau },b_{\tau }\right)$$

where $0_{K}$ represents a *K*-dimensional column vector with element 0, β is the accuracy parameter of the mean, $a_{\alpha }$, $b_{\alpha }$, $a_{\tau }$, $b_{\tau }$ are the hyper-parameters of the distributions over these RVs, and G(·) is the gamma distribution probability density function. Particularly, when little or no prior information is obtained for these priors, it is suggested to set noninformative priors for the RVs to minimize the effect on the posterior distribution. That is, the values of hyperparameters $a_{\alpha }$, $b_{\alpha }$, $a_{\tau }$, and $b_{\tau }$ are set as small as possible so that the posteriors do not rely on the priors, but only on the data.

**Remark** **1.** To facilitate model training, rewrite Equation (8) as $p\left(W|\alpha \right)=\prod_{k=1}^{K}N\left(w_{k}^{*}|0,A^{-1}\right)$, where $A=diag\left\{\alpha_{1},\alpha_{2},\|,\alpha_{M}\right\}$. Each inverse variance of the $w_{m}$ is controlled by the corresponding $\alpha_{m}$. Thus, if the posterior distribution of a particular $\alpha_{m}$ is focused on larger values, the corresponding $w_{m}$ will converge to be tiny, effectively deactivating this particular direction in the latent space. Therefore, the effective dimensionality of the potential space is determined automatically.

According to Figure 1, the equation shown as Equation (12) represents the joint probability density function between training data D and the RVs Θ as follows:

(12) p(D,Θ)=p(D∣Z,W,μ,τ)p(Z)p(W∣α)p(μ)p(α)p(τ).

The VI is employed to train the DVBPCA to obtain the posterior distributions q(Θ) of the RVs Θ={Z,W,μ,α,τ}.

Assume the posterior distribution over Θ, q(Θ), can factorize into

(13) q(Θ)=q(Z)q(W)q(μ)q(α)q(τ)

According to Equation (2), the posterior distribution $q^{*}\left(z_{n}\right)$ over $z_{n}$ is detailed as

(14) $$\begin{array}{cc} \ln q^{*}\left(Z\right) & =E_{W,\mu ,\alpha ,\tau }\left[\ln p\left(D,\Theta \right)\right]+C\\ \; & =E_{W,\mu ,\tau }\left[\ln p\left(D|Z,W,\mu ,\tau \right)\right]+E\left[p\left(Z\right)\right]+C\\ \; & =\sum_{n=1}^{N}E_{W,\mu ,\tau }\left[-\frac{\tau }{2}{\left(d_{n}-Wz_{n}-\mu \right)}^{T}\left(d_{n}-Wz_{n}-\mu \right)\right]-\sum_{n=1}^{N}E\left[\frac{1}{2}z_{n}^{T}z_{n}\right]+C\\ \; & =\sum_{n=1}^{N}\left\{\langle \tau \rangle {\left(d_{n}-\langle \mu \rangle \right)}^{T}\langle W\rangle z_{n}-\frac{1}{2}\left(\langle \tau \rangle \langle W^{T}W\rangle +I_{M}\right)z_{n}\right\}+C\\ & =\sum_{n=1}^{N}\ln N\left(z_{n}|m_{z}^{\left(n\right)},\Sigma_{z}\right) \end{array}$$

where $m_{z}^{\left(n\right)}=\langle \tau \rangle \Sigma_{z}\langle W^{T}\rangle \left(d_{n}-\langle \mu \rangle \right)$, $\;\Sigma_{z}={\left(\langle \tau \rangle \langle W^{T}W\rangle +I_{M}\right)}^{-1}$, and 〈·〉 is the expectation.

Similarly, by using the general rule given by Equation (2), we can find the variational posterior distributions over other RVs, as shown on the right side of Equation (13) one by one, which are given as follows.

The posterior distribution $q^{*}\left(W\right)$ over W is obtained as

(15) $$q^{*}\left(W\right)=\prod_{k=1}^{K}N\left(w_{k}^{*}|m_{w}^{\left(k\right)},\Sigma_{w}\right)$$

where $\Sigma_{w}={\left(\sum_{n=1}^{N}\langle \tau \rangle \langle z_{n}z_{n}^{T}\rangle +A\right)}^{-1}$, and $m_{w}^{\left(k\right)}=\sum_{n=1}^{N}\langle \tau \rangle \Sigma_{w}\langle z_{n}\rangle \left(d_{nk}-\langle \mu_{k}\rangle \right)$.

The posterior distribution $q^{*}\left(\mu \right)$ over μ is obtained as

(16) $$q^{*}\left(\mu \right)=N\left(\mu |m_{\mu },\Sigma_{\mu }\right)$$

where $\Sigma_{\mu }={\left(\langle \tau \rangle N+\beta \right)}^{-1}I_{K}$, and $m_{\mu }=\sum_{n=1}^{N}\langle \tau \rangle \Sigma_{\mu }\left(d_{n}-\langle W\rangle \langle z_{n}\rangle \right)$.

The posterior distribution $q^{*}\left(\alpha \right)$ over α is obtained as

(17) $$q^{*}\left(\alpha \right)=\prod_{m=1}^{M}G\left(\alpha_{m}|c_{\alpha },d_{\alpha }\right)$$

where $c_{\alpha }=\frac{K}{2}+a_{\alpha }$, and $d_{\alpha }=b_{\alpha }+\frac{1}{2}\langle w_{m}^{T}w_{m}\rangle$.

The posterior distribution $q^{*}\left(\tau \right)$ over τ is obtained as

(18) $$q^{*}\left(\tau \right)=G\left(\tau |c_{\tau },d_{\tau }\right)$$

where $d_{\tau }=b_{\tau }+\frac{1}{2}\sum_{n=1}^{N}\left\{d_{n}^{T}d_{n}-2d_{n}^{T}\left(\langle W\rangle \langle z_{n}\rangle +\langle \mu \rangle \right)+\langle z_{n}^{T}W^{T}Wz_{n}\rangle +2\langle z_{n}^{T}\rangle \langle W^{T}\rangle \langle \mu \rangle +\langle \mu^{T}\mu \rangle \right\}$, and $c_{\tau }=\frac{NK}{2}+a_{\tau }$.

The ELBO $L\left(q\right)$ is calculated using the updated posterior distributions, i.e.,

(19) $$\begin{array}{cc} L\left(q\right) & =E\left[\ln p\left(D,Z,W,\mu ,\alpha ,\tau \right)\right]-E\left[\ln q^{*}\left(Z,W,\mu ,\alpha ,\tau \right)\right]\\ \; & =E[\ln p(D|Z,W,\mu ,\tau )]+E[\ln p(Z)]+E[\ln p(W|\alpha )]+E[\ln p(\mu )]\\ \; & +E\left[\ln p\left(\alpha \right)\right]+E\left[\ln p\left(\tau \right)\right]-E\left[\ln q^{*}\left(Z\right)\right]-E\left[\ln q^{*}\left(W\right)\right]-E\left[\ln q^{*}\left(\mu \right)\right]\\ \; & -E\left[\ln q^{*}\left(\alpha \right)\right]-E\left[\ln q^{*}\left(\tau \right)\right] \end{array}$$

The convergence of the training procedure can be diagnosed with ELBO. The termination criteria for the parameter learning process are defined as detailed in Equation (20), as follows:

(20) $$|\frac{L^{iter+1}-L^{iter}}{L^{iter+1}}|<\delta$$

where $L^{iter+1}$ and $L^{iter}$ are the values of ELBO at the (iter+1)-th and iter-th iterative steps, respectively, and δ is the threshold.

The main steps in training the DVBPCA model are summarized in Algorithm 1.
**Algorithm 1** Pseudocode for the DVBPCA.  1:**Input:** The observed dataset D, *M*, β, tol, maximum number of iterations $Iter_{\max}$, prior hyper-parameters $\left(a_{\alpha },b_{\alpha },a_{\tau },b_{\tau }\right)$.    2:**Initialization:** initialize $m_{z}$, $\Sigma_{z}$, $m_{w}$, $\Sigma_{w}$, $m_{\mu }$, $\Sigma_{\mu }$, $c_{\alpha }$, $d_{\alpha }$, $c_{\tau }$, $d_{\tau }$.    3:**for** iter=1 to $Iter_{\max}$ **do**  4:    **VB-E-Step**     5:   Calculate $m_{z}$, $\Sigma_{z}$ using Equation (14).    6:    **VB-M-Step**    7:    Calculate $m_{w}$, $\Sigma_{w}$ using Equation (15).    8:    Calculate $m_{\mu }$, $\Sigma_{\mu }$ using Equation (16).    9:    Calculate $c_{\alpha }$, $d_{\alpha }$ using Equation (17).  10:    Calculate $c_{\tau }$, $d_{\tau }$ using Equation (18).  11:    Calculate L(q) using Equation (19).  12:    **if** the termination condition is satisfied **then**13:        **Break**  14:    **end if** 15:**end for** 16:**Output:** the posterior distributions of RVs Θ.

### 3.3. Moving Window-Based Dynamic Variational Bayesian Principal Component Analysis

The moving window (MW) method is used to trace time variations and reject disturbances [28]. It captures the latest process information by discarding the oldest sample after the latest sample is obtained and rebuilding a model with all data in the window. Specially, for the first local model, as mentioned above, set noninformation priors for the RVs. For subsequent local modeling, the prior distributions of the RVs in the MW-DVBPCA are updated one by one and replaced with the trained posterior distributions of the RVs at the previous moment. The MW-DVBPCA is detailed in this subsection.

Firstly, the training dataset (i.e., historical data) is preset in the MW, and the DVBPCA is trained on the training dataset to obtain the posterior distributions of the RVs Θ. When the test samples (i.e., online samples) are acquired, the DVBPCA generated earlier is used to estimate the KVs, and the posterior distributions of RVs are used as the prior for the new DVBPCA model, which is elaborated as follows.

Let $x_{n^{\prime }}$ and $y_{n^{\prime }}$ denote the observations of inputs and outputs at certain online time instant $n^{\prime }$, respectively, where $y_{n^{\prime }}$ is unknown. The hidden RVs $z_{n^{\prime }}$ are first introduced.

Since the outputs $y_{n^{\prime }}$ are unknown, the posterior distribution over $z_{n^{\prime }}$ is calculated with the observation of $x_{n^{\prime }}$ which, based on Equation (14), is given by the equation as shown in Equation (21) as follows:

(21) $$q^{*}\left(z_{n^{\prime }}\right)=N\left(z_{n}|m_{z}^{\left(n^{\prime }\right)},\Sigma_{z}^{n^{\prime }}\right)$$

where $\Sigma_{z}^{n^{\prime }}={\left(\langle \tau \rangle \langle W_{x}^{T}W_{x}\rangle +I_{M}\right)}^{-1}$, and $m_{z}^{\left(n^{\prime }\right)}=\langle \tau \rangle \Sigma_{z}^{n^{\prime }}\langle W_{x}^{T}\rangle \left(x_{n^{\prime }}-\langle \mu \rangle \right)$.

Then, the conditional distribution of $y_{n^{\prime }}$ given $x_{n^{\prime }}$ is obtained as

(22) $$\begin{array}{cc} \; & p\left(y_{n^{\prime }}|x_{n^{\prime }}\right)=\int p\left(y_{n^{\prime }}|z_{n^{\prime }},W_{y},\mu_{y},\tau \right)p\left(z_{n^{\prime }}|x_{q}\right)q^{*}\left(W_{y}\right)q^{*}\left(\mu_{y}\right)q^{*}\left(\tau \right)dzdWd\mu d\tau \\ \; & =\int N\left(y_{n^{\prime }}|W_{y}^{T}z_{n^{\prime }}+\mu_{y},\tau^{-1}I_{K}\right)N\left(z_{n^{\prime }}|m_{z}^{\left(n^{\prime }\right)},\Sigma_{z}^{n^{\prime }}\right)N\left(W_{y}|m_{w}^{y},\Sigma_{w}\right)N\left(\mu_{y}|m_{\mu }^{y},\Sigma_{\mu }^{yy}\right)G\left(\tau |c_{z},d_{z}\right)dzdWd\mu d\tau \end{array}$$

where $m_{w}^{y}$ and $\Sigma_{w}$ are the mean and covariance calculated from the posterior distribution $q^{*}\left(W_{y}\right)$, respectively, and $m_{\mu }^{y}$ and $\Sigma_{\mu }^{yy}$ are the mean values and covariance matrix calculated from the posterior distribution $q^{*}\left(\mu_{y}\right)$, respectively.

Equation (22) can be transformed into

(23) $$p\left(y_{n^{\prime }}|x_{n^{\prime }}\right)=\int N\left(y_{n^{\prime }}|m_{w}^{yT}m_{z}^{\left(n^{\prime }\right)}+m_{\mu }^{y},\mathrm{tr}\left[\Sigma_{w}\Sigma_{z}^{n^{\prime }}+\Sigma_{w}m_{z}^{\left(n^{\prime }\right)}m_{z}^{(n^{\prime })T}+m_{w}^{y}m_{w}^{yT}\Sigma_{z}^{n^{\prime }}\right]+\Sigma_{\mu }^{yy}+\tau^{-1}I_{K}\right)G\left(\tau |c_{\tau },d_{\tau }\right)d\tau$$

where tr[·] represents the trace.

The mean vector and covariance matrix of Gaussian distribution in Equation (23) are obtained as illustrated in Equation (24) and Equation (25), respectively, as follows:

(24) $$E_{p\left(y_{n^{\prime }}|x_{n^{\prime }},\tau \right)}\left[y_{n^{\prime }}\right]=E\left[W_{y}^{T}z_{n^{\prime }}+\mu_{y}+\varepsilon_{y}\right]=m_{w}^{yT}m_{z}^{\left(n^{\prime }\right)}+m_{\mu }^{y}$$

(25) $$\begin{array}{cc} V_{p\left(y_{n^{\prime }}|x_{n^{\prime }},\tau \right)}\left[y_{n^{\prime }}\right] & =V[W_{y}^{T}z_{n^{\prime }}+\mu_{y}+\varepsilon_{y}]\\ \; & =V\left[W_{y}^{T}z_{n^{\prime }}\right]+V\left[\mu_{y}\right]+V\left[\varepsilon_{y}\right]\\ \; & =E\left[W_{y}^{T}z_{n^{\prime }}z_{n^{\prime }}^{T}W_{y}\right]-m_{w}^{yT}m_{z}^{\left(n^{\prime }\right)}m_{z}^{(n^{\prime })T}m_{w}^{y}+\Sigma_{\mu }^{yy}+{\left(\tau \right)}^{-1}I_{K}\\ \; & =\mathrm{tr}\left(E\left[W_{y}W_{y}^{T}z_{n^{\prime }}z_{n^{\prime }}^{T}\right]-m_{w}^{y}m_{w}^{yT}m_{z}^{\left(n^{\prime }\right)}m_{z}^{(n^{\prime })T}\right)+\Sigma_{\mu }^{yy}+{\left(\tau \right)}^{-1}I_{K}\\ \; & =\mathrm{tr}\left[\Sigma_{w}\Sigma_{z}^{n^{\prime }}+\Sigma_{w}m_{z}^{\left(n^{\prime }\right)}m_{z}^{(n^{\prime })T}+m_{w}^{y}m_{w}^{yT}\Sigma_{z}^{n^{\prime }}\right]+\Sigma_{\mu }^{yy}+\Sigma_{\mu }^{yy}+{\left(\tau \right)}^{-1}I_{K} \end{array}$$

where $p\left(\varepsilon_{y}\right)=N\left(\varepsilon_{y}|0,{\left(\tau \right)}^{-1}I_{K}\right)$.

By replacing the distribution of τ with its expectation, the probability distribution of the predicted KVs is approximated as

(26) $$p\left(y_{n^{\prime }}|x_{n^{\prime }}\right)\approx N\left(y_{n^{\prime }}|m_{w}^{yT}m_{z}^{\left(n^{\prime }\right)}+m_{\mu }^{y},\mathrm{tr}\left[\Sigma_{w}\Sigma_{z}^{n^{\prime }}+\Sigma_{w}m_{z}^{\left(n^{\prime }\right)}m_{z}^{(n^{\prime })T}+m_{w}^{y}m_{w}^{yT}\Sigma_{z}^{n^{\prime }}\right]+\Sigma_{\mu }^{yy}+{\left(\frac{c_{\tau }}{d_{\tau }}\right)}^{-1}I_{K}\right).$$

Therefore, based on Equation (26), the estimations ${\hat{y}}_{n^{\prime }}$ of $y_{n^{\prime }}$ are given by

(27) $${\hat{y}}_{n^{\prime }}=E_{p\left(y_{n^{\prime }}|x_{n^{\prime }}\right)}\left[y_{n^{\prime }}\right]=m_{w}^{yT}m_{z}^{\left(n^{\prime }\right)}+m_{\mu }^{y}$$

and the prediction uncertainty is quantified by the variance matrix of $y_{n^{\prime }}$ denoted by $V_{p\left(y_{n^{\prime }}|x_{n^{\prime }}\right)}\left[y_{n^{\prime }}\right]$, which is given by Equation (28), i.e.,

(28) $$V_{p\left(y_{n^{\prime }}|x_{n^{\prime }}\right)}\left[y_{n^{\prime }}\right]=\mathrm{tr}\left[\Sigma_{w}\Sigma_{z}^{n^{\prime }}+\Sigma_{w}m_{z}^{\left(n^{\prime }\right)}m_{z}^{(n^{\prime })T}+m_{w}^{y}m_{w}^{yT}\Sigma_{z}^{n^{\prime }}\right]+\Sigma_{\mu }^{yy}+{\left(\frac{c_{\tau }}{d_{\tau }}\right)}^{-1}I_{K}.$$

Once the KVs predictions for the new sample are completed, the historical data window is slid down to include the latest measured sample set $\left\{x_{n^{\prime }},y_{n^{\prime }}\right\}$ and to eliminate the oldest sample. The DVBPCA is then retrained according to the current window of the historical dataset for online prediction. This process repeats once new samples are obtained online.

### 3.4. Differential Evolutionary-Based Model Selection

The DE is an effective and simple intelligent optimization algorithm commonly used for solving NP-hard problems in real number fields [29]. Associated studies have demonstrated that DE has fast convergence performance as an excellent global optimization algorithm. Therefore, the DE is utilized for model selection (i.e., selecting the best parameter) of the DVBPCA and MW-DVBPCA, involving the dynamic orders p and time delays l given in Equation (4). Equation (29) shows the optimization problem, as follows:

(29) $$\begin{array}{cc} \; & \min\;g(p,l)\\ \; & s.t.\;\;p_{\min}\leq p\leq p_{\max}\\ \; & \;\;\;\;\;\;\;l_{\min}\leq l\leq l_{\max} \end{array}$$

where g(·) is the fitness function, $p_{\min}$ and $p_{\max}$ are the lower and upper bounds of the dynamic orders p, respectively, and $l_{\min}$ and $l_{\max}$ are the lower and upper bounds of the delays l, respectively.

Based on Equation (4), {p,l} are non-negative integers. Therefore, the individuals generated by the DE need to be rounded. And the DE algorithm contains three core evolutionary operations, i.e., mutation, crossover and selection, which are introduced in detail as follows.

*Initialization.* Set the population size NP, dimension of individual (2S), and the upper bounds $\left(\rho_{1}^{\left(max\right)},\rho_{2}^{\left(max\right)},\cdots ,\rho_{2S}^{\left(max\right)}\right)$ and the lower bounds $\left(\rho_{1}^{\left(min\right)},\rho_{2}^{\left(min\right)},\cdots ,\rho_{2S}^{\left(min\right)}\right)$ of the dynamic orders and time delays, where individuals $\left(\rho_{1},\rho_{2},\cdots ,\rho_{2S}\right)$ in the population are randomly generated in integer field by Equation (30), i.e.,

(30) $$\rho_{e}=\rho_{e}^{\left(min\right)}+\left(\rho_{e}^{\left(max\right)}-\rho_{e}^{\left(min\right)}\right)\cdot \mathrm{rand}\left(NP,1\right)$$

*Mutation.* In the *r*-th generation, DE generates a mutation vector $v_{e}$ for the *e*-th individual, given by Equation (31); that is,

(31) $$v_{e}^{r+1}=\rho_{e_{0}}^{r}+F\cdot \left(\rho_{e_{1}}^{r}-\rho_{e_{2}}^{r}\right)$$

where $\rho_{e_{0}}^{r}$, $\rho_{e_{1}}^{r}$, and $\rho_{e_{2}}^{r}$ are individuals that randomly selected from population with $e_{0}\neq e_{1}\neq e_{2}\neq e$, and *F* is the mutation rate.

*Crossover.* A trial individual vector $u_{e}$ is generated by the original individual vector or the mutation vector based on the crossover rate CR ranged in (0,1), which is shown in Equation (32), i.e.,

(32) $$u_{i,e}^{r+1}=\left\{\begin{array}{cc} v_{i,e}^{r+1}, & \;\mathrm{if}\;\mathrm{rand}\left(0,1\right)\leq CR\;\mathrm{or}\;e={\mathrm{rand}}_{e}\\ \rho_{i,e}^{r}, & \;\mathrm{otherwise}\; \end{array}\right.$$

where ${\mathrm{rand}}_{e}$ is a random integer ranged in (1,2S).

*Selection.* According to the fitness function g(·), select the individual with a lower value as detailed in Equation (33) as follows:

(33) $$\rho_{e}^{r+1}=\left\{\begin{array}{cc} u_{e}^{r+1}, & \;\mathrm{if}\;g\left(u_{e}^{r+1}\right)\leq g\left(\rho_{e}^{r}\right)\\ \rho_{e}^{r}, & \;\mathrm{otherwise}\; \end{array}\right.$$

*Termination.* The evolution continues until it reaches the given maximum generation or the change in the fitness value is small. Otherwise, the above three operators should be performed repeatedly.

**Figure 2:**
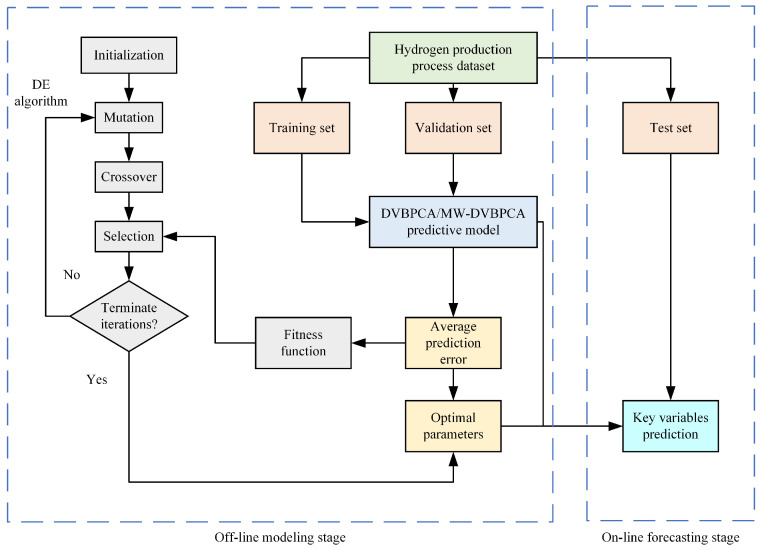
The flowchart of the DVBPCA and MW-DVBPCA.

Figure 2 shows the flowchart of the DVBPCA and MW-DVBPCA combined with the DE for parameter optimization. Note that for the multi-output models, DVBPCA and MW-DVBPCA, the average prediction error of four KVs is set as the fitness function.

## 4. Case Studies and Comparisons

A comparative study of data-driven virtual sensors upon a real-life natural gas steam reforming hydrogen production process is provided in this section. Meanwhile, a case study is useful for understanding the hydrogen production processes and data features. The state-of-the-art virtual sensors and developed models (DVBPCA and MW-DVBPCA) are investigated, including partial least squares (PLS) [30], VBPCA, long short-term memory (LSTM) [31], echo state networks (ESN) [32], and dynamic PLS (DPLS) [33]. The PLS is a classic static model with the advantages of modeling data colinearities and simple data structure. The VBPCA is a static probabilistic model. Different from the PLS, the VBPCA determines the number of principal components automatically, which is also the basis of our developed models (DVBPCA and MW-DVBPCA). The LSTM and ESN are advanced dynamic neural network models that consider the dynamics. The DPLS is the improvement of the PLS based on the FIR paradigm, which also accounts for the dynamics of the hydrogen production process.

### 4.1. Natural Gas Steam Reforming Hydrogen Production Process

**Figure 3:**
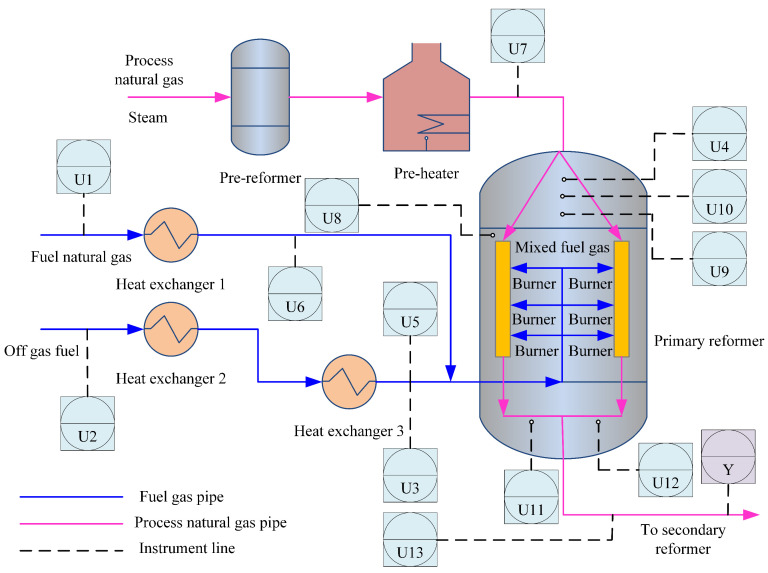
The flowchart of steam reforming process.

The natural gas steam reforming hydrogen production process is the largest industrial source of hydrogen, and it consists of four main processes: feedstock purification, steam reforming, medium temperature conversion, and pressure swing adsorption. Among these processes, steam reforming is the dominant reaction process, which is schematically shown in Figure 3 [34]. Processed gases are mixed with steam in the pre-reformer, where all hydrocarbons and some ${\mathrm{CH}}_{4}$ are converted to CO, ${\mathrm{CO}}_{2}$ and $H_{2}O$. The temperature then decreases, which is not conducive to promoting hydrogen generation. The pre-reformer output is heated in a pre-heater and then continuously fed to the primary reformer for complete reforming.

The main chemical reactions of this process are shown as follows:

(34) $$\begin{array}{cc} C_{n}H_{2n+2}+nH_{2}O & \overset{\Delta }{⟷}n\mathrm{CO}+\left(2n+1\right)H_{2}\\ {\mathrm{CH}}_{4}+H_{2}O & \overset{\Delta }{⟷}\mathrm{CO}+3H_{2}\\ \mathrm{CO}+H_{2}O & \overset{\Delta }{⟷}{\mathrm{CO}}_{2}+H_{2}. \end{array}$$

According to Equation (34), the exit gases of the hydrogen production process consist of ${\mathrm{CH}}_{4}$, CO, ${\mathrm{CO}}_{2}$, and $H_{2}$, and the concentrations of these gases are KVs (as labeled “Y” in Figure 3) related to product quality. Therefore, these KVs need to be strictly monitored. In practice, offline laboratory analysis and hardware sensors are traditional methods for measuring the KVs, but have delays and high investment. Meanwhile, series-wound devices (such as pre-reformers and pre-heaters) introduce considerable transportation delays, which must be considered in virtual sensor modeling. Therefore, for giving real-time predictions of the KVs, a virtual sensor considering transportation delays is desirable.

### 4.2. Explanatory Variable Selection and Data Collection

A total of 13 EVs for the virtual sensor development are selected based on the first principles and the operation experience of the engineers. These EVs are easily measured variables, closely linked to the KVs and highly correlated with process variations, as shown by labels U1–U13 in Figure 3. The descriptions of these EVs are listed in Table 1.

**Figure 4:**
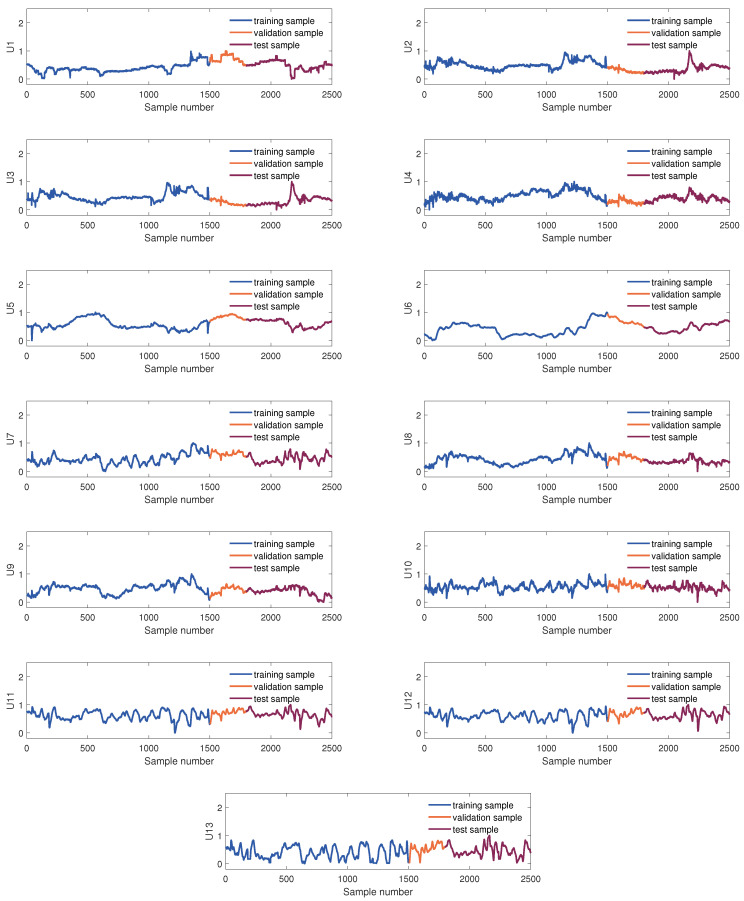
The diagram of data partition of 13 EVs.

**Figure 5:**
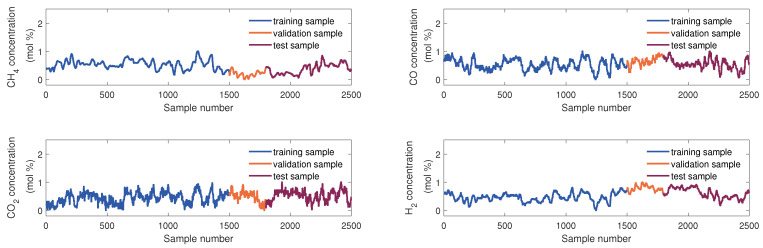
The diagram of data partition of four KVs.

The samples used to develop the virtual sensor of the KVs were obtained from the DCS database. The KVs’ sampling rate is 10 min, and 2500 samples were collected. These were divided into three parts in order of collection time: the training set, the validation set, and the testing set, which consist of 1500 samples, 300 samples, and 700 samples, respectively. Figure 4 and Figure 5 show sample division and the changing trend of these EVs and four KVs, respectively. In this article, we use scaled dimensionless data for modeling.

### 4.3. Evaluation Metrics

The root mean squares error (RMSE), the coefficient of determination ($R^{2}$), and the mean absolute error (MAE) are chosen to quantify different models’ performance, which are defined as

(35) $$\begin{array}{cc} \; & \mathrm{RMSE}=\sqrt{\frac{1}{N^{\prime }}\sum_{n^{\prime }=1}^{N^{\prime }}{\left({\hat{y}}_{n^{\prime }}-y_{n^{\prime }}\right)}^{2}}\\ \; & R^{2}=1-\sum_{n^{\prime }=1}^{N^{\prime }}{\left({\hat{y}}_{n^{\prime }}-y_{n^{\prime }}\right)}^{2}/\sum_{n^{\prime }=1}^{N^{\prime }}{\left(y_{n^{\prime }}-\bar{y}\right)}^{2}\\ \; & \mathrm{MAE}=\frac{1}{N^{\prime }}\sum_{n^{\prime }=1}^{N^{\prime }}\left|{\hat{y}}_{n^{\prime }}-y_{n^{\prime }}\right| \end{array}$$

where ${\hat{y}}_{n^{\prime }}$ and $y_{n^{\prime }}$ are the estimated values and true values of the $n^{\prime }$th testing sample, respectively, $N^{\prime }$ is the size of the testing dataset, and $\bar{y}=\sum_{n^{\prime }=1}^{N^{\prime }}y_{n^{\prime }}/N^{\prime }$ are the mean of the KVs in the testing dataset. The RMSE and MAE indexes characterize the average and largest prediction errors on the test set, respectively, and the $R^{2}$ index characterizes the correlation between the predicted and true values. Therefore, the smaller the RMSE and MAE or the bigger the $R^{2}$, the higher the predictive accuracy.

### 4.4. Parameter Selection

All models’ optimal parameters need to be chosen to obtain different models’ best prediction performance. Select the DE algorithm to minimize the average RMSE (i.e., the mean of the RMSE of the four KVs) on the validation set for parameter optimization of different models. For the PLS, the number of principal components and the time delays are the parameters to be optimized. For the DPLS, the number of principal components, the time delays and the dynamic orders are the parameters to be optimized. For the VBPCA-based models, the time delays and the dynamic orders are the parameters to be optimized. For the LSTM and ESN, the time delays are the parameters to be optimized.

**Figure 6:**
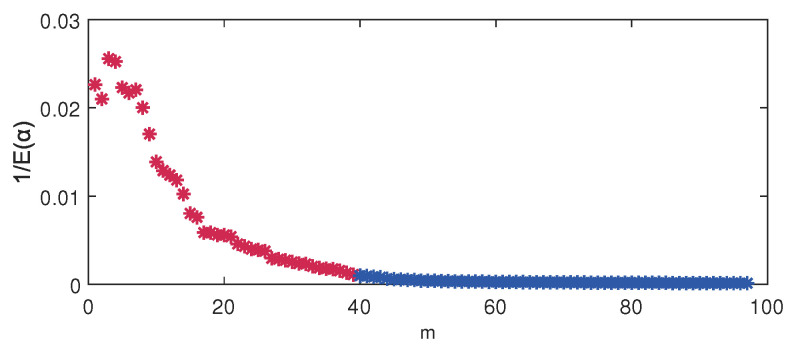
Variance of each $w_{m}$ by DVBPCA.

For the VBPCA-based models, the key issue of automatic parameter determination of the principal component number is whether each column of the loading matrix W is either insignificant or significant, i.e., $w_{m}=0$ or $w_{m}\neq 0$, which can be controlled by α according to Equation (8). Take DVBPCA for example, the fact that the variance of each $w_{m}$ can be quantified by $1/E(\alpha_{m})$ for m=1,‖,M, which is displayed in Figure 6. The color distinction of the points in Figure 6 means the value of $1/E(\alpha_{m})$ is either insignificant or significant by setting threshold, where red means significant and blue means insignificant. As shown in Figure 6, the appropriate dimensionality of the principal component subspace is selected as 39.

For the ESN, set the input regulation scale to 0.1, set the reservoir size to 50, and set the spectral radius to 0.8. Considering that the ESN involves the random weights in the reservoir computing step, 20 modeling tests were performed. For each trial, the random weights are saved and fixed, and the DE algorithm is used to optimize the model delays further. Then, the best results from 20 tests are picked for model performance comparison. Note that the ESN is a single-output model; we therefore construct four ESN models, one for each KV. For the LSTM, based on the debugging experience, set the time step to 1, set the learning rate to 0.1, set the hidden layer to 1, and set the neuron number in the hidden layer to 100, which performs favorably empirically in each of the replicated experiments of this work.

**Figure 7:**
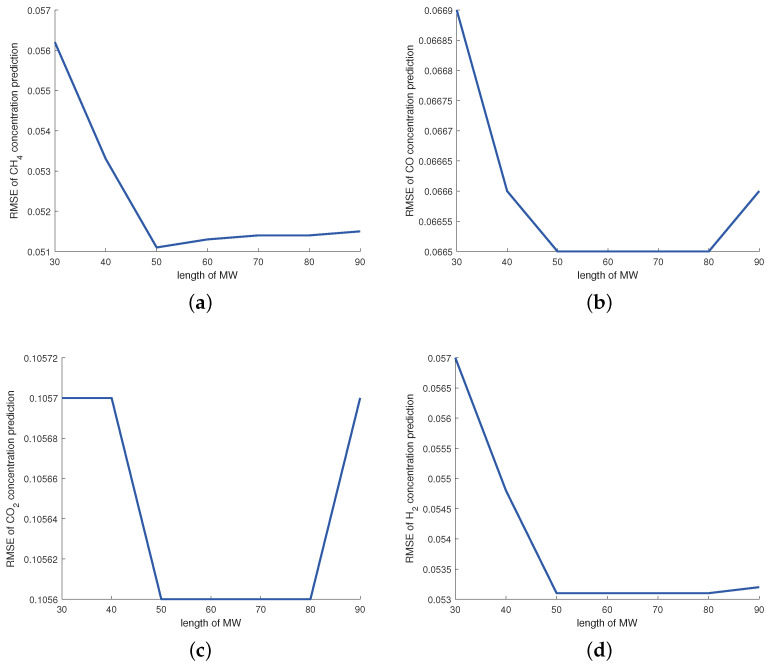
Impact of MW size for: (**a**) ${\mathrm{CH}}_{4}$ concentration, (**b**) CO concentration, (**c**) ${\mathrm{CO}}_{2}$ concentration, and (**d**) $H_{2}$ concentration.

For the MW-DVBPCA, the impact of MW size is detailed in Figure 7. The MW size is in units of 10 min, i.e., setting the MW size to 50 means that the MW contains 500 min of data. As shown in Figure 7, for small MW sizes, the data in the window may not appropriately represent the relationship between process variables. In contrast, excessive MW size covers too much outdated sample data so that the MW-DVBPCA fails to track the process change adequately. Therefore, according to Figure 7, the MW size was selected as 50.

### 4.5. Results and Analysis

**Figure 8:**
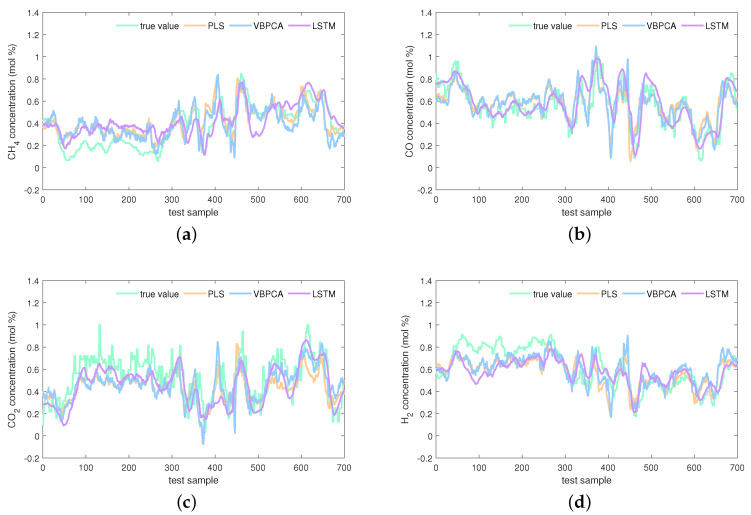
Estimations of four KVs by the PLS, VBPCA, and LSTM: (**a**) ${\mathrm{CH}}_{4}$ concentration, (**b**) CO concentration, (**c**) ${\mathrm{CO}}_{2}$ concentration, and (**d**) $H_{2}$ concentration.

**Figure 9:**
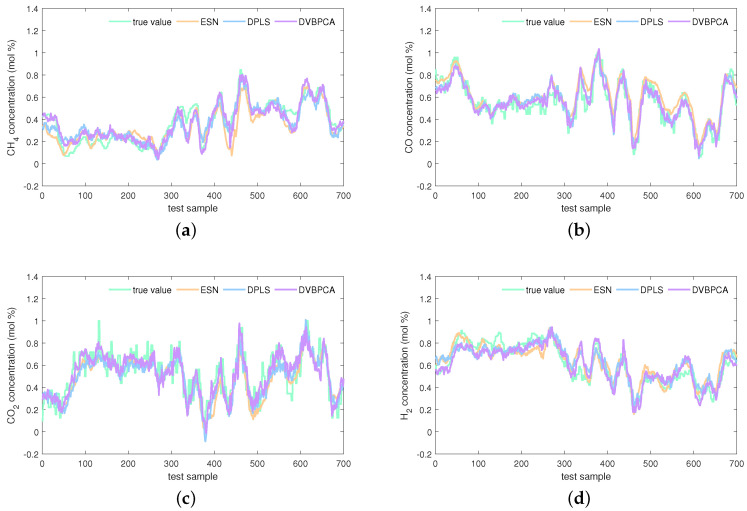
Estimations of four KVs by the ESN, DPLS, and DVBPCA: (**a**) ${\mathrm{CH}}_{4}$ concentration, (**b**) CO concentration, (**c**) ${\mathrm{CO}}_{2}$ concentration, and (**d**) $H_{2}$ concentration.

**Figure 10:**
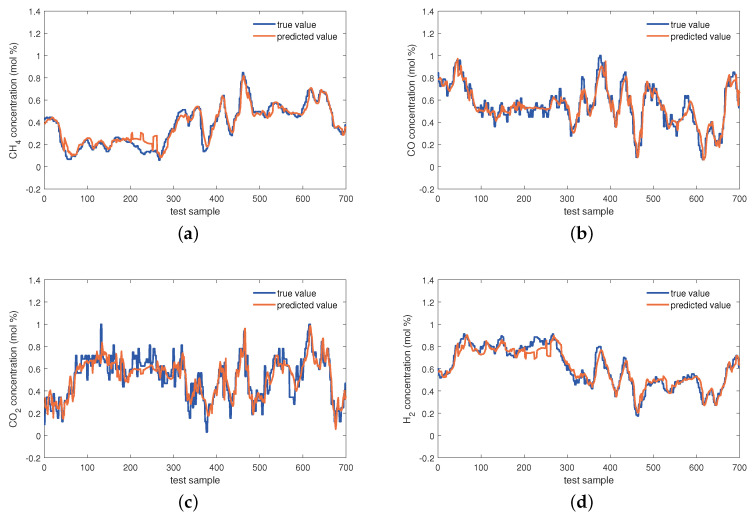
Estimations of four KVs by the MW-DVBPCA: (**a**) ${\mathrm{CH}}_{4}$ concentration, (**b**) CO concentration, (**c**) ${\mathrm{CO}}_{2}$ concentration, and (**d**) $H_{2}$ concentration.

The estimations on the test set of the KVs obtained by the investigated seven virtual sensors are visualized in Figure 8, Figure 9 and Figure 10. In Figure 8, obviously, the PLS and VBPCA have the poor estimation performance. Because of the significant dynamics of the hydrogen production process, the estimation accuracy of the static models, PLS and VBPCA, is not as satisfactory as that of the other five dynamic models (such as around the 120-th sample of the ${\mathrm{CO}}_{2}$ concentration). Figure 9 shows that the estimated values of the DVBPCA tracks real values better than the other three models (such as around the 300-th sample of the CO concentration and around the 300-th sample of the ${\mathrm{CO}}_{2}$ concentration). That is because, on the one hand, the DVBPCA can deal with the colinearities between the EVs compared with the LSTM and ESN. On the other hand, the DVBPCA can tackle the overfitting results from high-order variable augmentation compared with the DPLS. Moreover, the ESN constructs four virtual sensors, one for each KV for estimation, but the DVBPCA is a multi-output model that considers the inherent relationships between the KVs. Figure 10 illustrate that the estimations of the four KVs by the MW-DVBPCA match the real values much better (particularly in the localized area of the ${\mathrm{CH}}_{4}$ concentration around the 440-th sample) than other models, revealing the importance of considering time variation properties in the virtual sensor modeling of the hydrogen production process. Moreover, although the overall predicted values do match well with the true ones when the proposed MW-DVBPCA method was used, some discrepancies can be observed between test sample number 200 and 300 for all the four KVs. The possible reasons for these differences in the predicted and true values are as follows. Firstly, the characteristics of the samples between test sample number 200 and 300 are changing rapidly, and model learning does not accommodate such changes in time. Secondly, the samples between test sample number 200 and 300 have nonlinearities, but the local model constructed by MW-DVBPCA is linear. Overall, it is recognized that the proposed models show noticeable advantages over the benchmark models.

**Figure 11:**
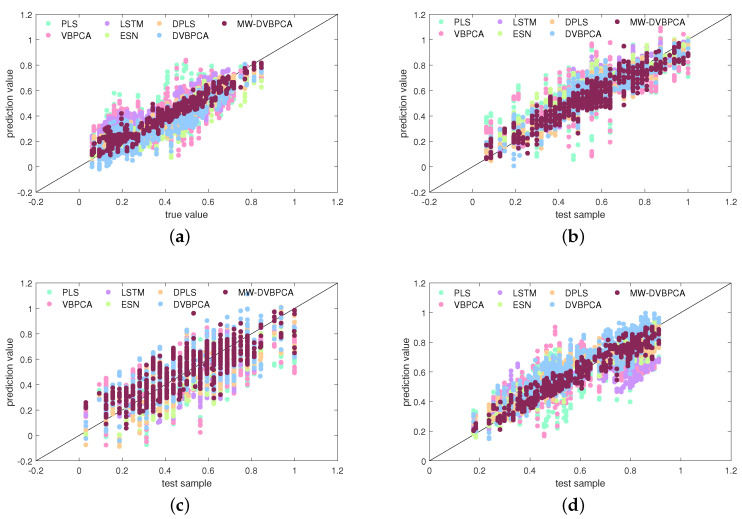
Scatter plot comparisons between various models for: (**a**) ${\mathrm{CH}}_{4}$ concentration, (**b**) CO concentration, (**c**) ${\mathrm{CO}}_{2}$ concentration, and (**d**) $H_{2}$ concentration.

Figure 11 compares the seven models in terms of scatter plots. Based on Figure 11, a further comparison of the prediction of the data-driven models can be made. As shown in Figure 11a, the predictions of the PLS for ${\mathrm{CH}}_{4}$ component deviate obviously from the real values. Figure 11b,c shows that the VBPCA model improves the prediction accuracy of CO and ${\mathrm{CO}}_{2}$ concentrations somewhat compared to the PLS model. However, the overall prediction accuracy is still very low. Figure 11d reveals that all models have relatively poor prediction accuracy for the $H_{2}$ concentration, but the MW-DVBPCA presents a better result. As indicated in Figure 11, the scatters by MW-DVBPCA are more closely and clearly located around the diagonal line than those of other models, thus illustrating better performance.

The estimation performance of all data-driven virtual sensors is quantitatively tabulated in Table 2 and Table 3. For a further comparison, Table 2 and Table 3 show the estimations of the data-driven virtual sensors not considering transportation delays. These models’ parameter selection is consistent with the corresponding model considering transportation delays. Overall, the estimation results in Table 2 and Table 3 provide an initial validation of the effectiveness of the data-driven virtual sensors. However, the performances of the different data-driven virtual sensors vary considerably. The performance of the models that consider delays is better than that of the corresponding models that do not, as shown in Table 2 and Table 3. Take the DVBPCA as an instance. The predictive performance based on the RMSE index of the four KVs by the model accounting for the time delays is improved by 18.6%, 13.0%, 2.3%, and 5.5%, respectively, compared to the model ignoring time delays. This is because the hydrogen production process has substantial time delays, and it has been proven that ignoring the delays could result in significantly deteriorated performance [29]. The $R^{2}$ values for two static models, i.e., the PLS and VBPCA, are as low as below 0.5; in contrast, the dynamic models, such as LSTM, ESN, and DPLS, show significantly better performance than the PLS and VBPCA, indicating the dynamic model better fits the data features of the actual hydrogen production process. Moreover, due to the capability of dealing with overfitting, the DVBPCA performs better than the DPLS. Concretely, compared to the DPLS, the RMSEs of the four KVs obtained by the DVBPCA are decreased by 13.4%, 1.8%, 3.2%, and 4.8%, respectively. Moreover, the MW-DVBPCA further improves the estimations of the four KVs. The $R^{2}$s of the ${\mathrm{CH}}_{4}$ and $H_{2}$ concentrations reach as high as up to 0.9. Compared with the DVBPCA, the predictive performance on the four KVs by the MW-DVBPCA improves by 1.3%, 10.3%, 2.8%, and 33.4%, respectively, in terms of the MAE index.

Furthermore, to check whether the MW-DVBPCA’s performance is significantly different from that of other models, the Wilcoxon test is employed for statistical testing [35]. The Wilcoxon test is a non-parametric testing method which is used to examine whether there is significant difference in the median values of the squared estimation errors obtained by the two virtual sensors. In Wilcoxon’s test, the likelihood that the corresponding hypothesis will be accepted is measured by calculating the *p*-value. The smaller the value of *p*-value, the lower the probability that the corresponding hypothesis will be accepted. Typically, the hypothesis should be rejected if the *p*-value is less than the given significance level η, the hypothesis should be rejected; that is, statistically the median values of two virtual sensors are different.

The Wilcoxon test results are given in Table 4, where ${\bar{E}}_{\mathrm{PLS}}^{2}$, ${\bar{E}}_{\mathrm{VBPCA}}^{2}$, ${\bar{E}}_{\mathrm{LSTM}}^{2}$, ${\bar{E}}_{\mathrm{ESN}}^{2}$, ${\bar{E}}_{\mathrm{DPLS}}^{2}$, ${\bar{E}}_{\mathrm{DVBPCA}}^{2}$, and ${\bar{E}}_{\mathrm{MW}-\mathrm{DVBPCA}}^{2}$ mean the median values of squared estimated errors obtained by the PLS, VBPCA, LSTM, ESN, DPLS, DVBPCA, and MW-DVBPCA, respectively. Additionally, set the significance level η at 5%. As shown in Table 4, all hypothesized *p*-values are far below η. Hence, all hypotheses are rejected. In other words, there is statistical significance in comparing the MW-DVBPCA with other virtual sensors in the hydrogen production process.

### 4.6. Computational Efficiency Analysis

Since this article is concerned with real-time estimation, examining the runtime of the model is desirable. The offline and online computational efficiency of the virtual sensors is evaluated using the average over 10 independent simulations, including the CPU time consumed offline for parameter optimization (${\mathrm{CPT}}^{\mathrm{opt}}$) and the CPU time consumed online (${\mathrm{CPT}}^{\mathrm{online}}$). All experiments were computed on a Core i5 (2.90 GHz × 2) with 8 GB RAM, Windows 10 and R2021a version of MATLAB.

Table 5 lists the time taken by each virtual sensor on parameter determination. As can be observed, the ${\mathrm{CPT}}^{\mathrm{opt}}$ index for the DVBPCA model is much smaller than that for the LSTM, due to its more concise structure. The ${\mathrm{CPT}}^{\mathrm{opt}}$ indices for other dynamic global models are almost the same as those for the DVBPCA, but other dynamic global models have lower accuracy than the DVBPCA given in Table 2 and Table 3. Note that the ${\mathrm{CPT}}^{\mathrm{opt}}$ index for the MW-DVBPCA is much larger than that for the DVBPCA, which is because the MW-DVBPCA needs to rebuild the model each time it predicts a new valid sample. Fortunately, the parameter determination processes are carried out offline. In other words, this process hardly affects the online calculative efficiency of the MW-DVBPCA. The last column of Table 5 illustrates the online computation time of MW-DVBPCA. Consequently, the online computational efficiency of the developed models is not an issue. In practice, the ${\mathrm{CPT}}^{\mathrm{opt}}$ indices for all virtual sensors are less than 0.1 s/sample, significantly faster than the minimum sampling period for the KVs in the hydrogen production process. The results show that all data-driven virtual sensors meet the time requirements for real-time estimation, including the developed DVBPCA and MW-DVBPCA.

## 5. Conclusions and Outlook

Considering the complicated properties of the hydrogen production process, we develop virtual sensors for the KVs of the hydrogen production process in this article. The MW-DVBPCA is developed to model complicated properties of the hydrogen production process, including dynamics, time variations, and transportation delays. To be specific, the FIR paradigm and MW technique are employed to extract process dynamics and to deal with time variations, respectively. The time delays are determined automatically by the DE. A comparative study of developed virtual sensors and other state-of-the-art virtual sensors is carried out. Meanwhile, the performance of MW-DVBPCA is demonstrated by the real-life natural gas steam reforming hydrogen production process.

From the industrial point of view, the online estimations of the KVs of the hydrogen production process need further research. Some future works are given to further improve the estimation performance of the KVs.

- Robust methods. The probabilistic model in this article is based on the traditional Gaussian distribution assumption, which is susceptible to outliers. Therefore, the training set must be cleaned to remove outliers. However, some outliers are indistinctive and challenging to detect and remove. To this end, finding a probability distribution insensitive to the noise and outliers can help improve the generalization performance of predictive models. Typically, Student’s t distribution with heavier tails is a candidate choice. As a result, designing a robust virtual sensor based on Student’s t distribution is worth investigating.
- Data-driven approaches fused with process knowledge. In fact, the states (or the hidden variables) of the system are influenced by variables characterizing materials and energies fed into the process. Conventional virtual sensors take all observed variables as inputs and the KVs as outputs, which makes it difficult to describe the true causality between variables of the hydrogen production process, weakening the interpretability and generalization abilities. A causal virtual sensor can better reflect the process mechanism and thus estimate the KVs more accurately. Therefore, equipping the MW-DVBPCA model with the causality of process variables of the hydrogen production process is desirable.

## Figures and Tables

**Table 1:** EVs in the hydrogen production process.

EV	Description
U1	Flow of fuel natural gas into primary reformer
U2	Flow of fuel off gas into primary reformer
U3	Pressure of fuel off gas at the exit of heat exchanger 3
U4	Pressure of furnace flue gas at primary reformer’s exit
U5	Temperature of fuel off gas at the exit of heat exchanger 3
U6	Temperature of fuel natural gas at pre-heater’s exit
U7	Temperature of process gas at primary reformer’s entrance
U8	Temperature of furnace flue gas at primary reformer’s top left
U9	Temperature of furnace flue gas at primary reformer’s top right
U10	Temperature of mixed furnace flue gas at primary reformer’s top
U11	Temperature of transformed gas at primary reformer’s left exit
U12	Temperature of transformed gas at primary reformer’s right exit
U13	Temperature of transformed gas at primary reformer’s exit

**Table 2:** Quantitative estimation results of various models for the concentrations of ${\mathrm{CH}}_{4}$ and CO.

		${\mathrm{CH}}_{4}$			CO		
		RMSE	$R^{2}$	MAE	RMSE	$R^{2}$	MAE
➀	PLS	0.1915	−0.1329	0.1643	0.1944	−0.0975	0.1483
	VBPCA	0.2061	−0.2566	0.1703	0.2054	−0.2259	0.1592
	LSTM	0.1567	0.2733	0.1373	0.1351	0.5370	0.1080
	ESN	0.1150	0.6088	0.0965	0.1148	0.6173	0.0968
	DPLS	0.0942	0.7373	0.0770	0.0956	0.7348	0.0764
	DVBPCA	0.0827	0.7975	0.0704	0.0826	0.8018	0.0662
	MW-DVBPCA	0.0525	0.9186	0.0388	0.0709	0.8538	0.0533
➁	PLS	0.1348	0.4623	0.1077	0.1352	0.4687	0.1067
	VBPCA	0.1339	0.4694	0.1092	0.1315	0.4976	0.1052
	LSTM	0.1227	0.5543	0.1051	0.0829	0.8128	0.0606
	ESN	0.0955	0.7301	0.0759	0.0912	0.7582	0.0730
	DPLS	0.0777	0.8215	0.0647	0.0732	0.8444	0.0587
	DVBPCA	0.0673	0.8659	0.0536	0.0719	0.8497	0.0574
	MW-DVBPCA	0.0511	0.9228	0.0368	0.0665	0.8717	0.0515

➀ The time delays are not considered, ➁ the time delays are considered.

**Table 3:** Quantitative estimation results of various models for the concentrations of ${\mathrm{CO}}_{2}$ and $H_{2}$.

		${\mathrm{CO}}_{2}$			$H_{2}$		
		RMSE	$R^{2}$	MAE	RMSE	$R^{2}$	MAE
➀	PLS	0.1930	0.0095	0.1536	0.1882	−0.0729	0.1612
	VBPCA	0.1913	0.0264	0.1521	0.1973	−0.1792	0.1660
	LSTM	0.1562	0.3949	0.1293	0.1302	0.4863	0.1118
	ESN	0.1152	0.6471	0.0946	0.1057	0.6616	0.0895
	DPLS	0.1346	0.5185	0.1099	0.0955	0.7237	0.0779
	DVBPCA	0.1145	0.6514	0.0906	0.0796	0.8080	0.0676
	MW-DVBPCA	0.1103	0.6765	0.0858	0.0548	0.9090	0.0425
➁	PLS	0.1724	0.2094	0.1400	0.1320	0.4722	0.1090
	VBPCA	0.1648	0.2777	0.1361	0.1289	0.4964	0.1065
	LSTM	0.1352	0.5137	0.1094	0.1292	0.4943	0.1015
	ESN	0.1230	0.5978	0.1007	0.0823	0.7948	0.0678
	DPLS	0.1156	0.6448	0.0934	0.0790	0.8107	0.0669
	DVBPCA	0.1119	0.6670	0.0859	0.0752	0.8285	0.0605
	MW-DVBPCA	0.1056	0.7036	0.0835	0.0531	0.9146	0.0403

➀ The time delays are not considered, ➁ the time delays are considered.

**Table 4:** Results of the Wilcoxon test.

KV	Hypothesis	p-Value	Decision
${\mathrm{CH}}_{4}$ concentration	$H_{0}:{\bar{E}}_{\mathrm{MW}-\mathrm{DVBPCA}}^{2}={\bar{E}}_{\mathrm{PLS}}^{2}$	$2.79\times 10^{-70}$	$\;\mathrm{Reject}\;H_{0}$
	$H_{0}:{\bar{E}}_{\mathrm{MW}-\mathrm{DVBPCA}}^{2}={\bar{E}}_{\mathrm{VBPCA}}^{2}$	$1.48\times 10^{-71}$	$\;\mathrm{Reject}\;H_{0}$
	$H_{0}:{\bar{E}}_{\mathrm{MW}-\mathrm{DVBPCA}}^{2}={\bar{E}}_{\mathrm{LSTM}}^{2}$	$1.15\times 10^{-81}$	$\;\mathrm{Reject}\;H_{0}$
	$H_{0}:{\bar{E}}_{\mathrm{MW}-\mathrm{DVBPCA}}^{2}={\bar{E}}_{\mathrm{ESN}}^{2}$	$5.39\times 10^{-42}$	$\;\mathrm{Reject}\;H_{0}$
	$H_{0}:{\bar{E}}_{\mathrm{MW}-\mathrm{DVBPCA}}^{2}={\bar{E}}_{\mathrm{DPLS}}^{2}$	$6.32\times 10^{-30}$	$\;\mathrm{Reject}\;H_{0}$
	$H_{0}:{\bar{E}}_{\mathrm{MW}-\mathrm{DVBPCA}}^{2}={\bar{E}}_{\mathrm{DVBPCA}}^{2}$	$1.03\times 10^{-56}$	$\;\mathrm{Reject}\;H_{0}$
CO concentration	$H_{0}:{\bar{E}}_{\mathrm{MW}-\mathrm{DVBPCA}}^{2}={\bar{E}}_{\mathrm{PLS}}^{2}$	$7.67\times 10^{-45}$	$\;\mathrm{Reject}\;H_{0}$
	$H_{0}:{\bar{E}}_{\mathrm{MW}-\mathrm{DVBPCA}}^{2}={\bar{E}}_{\mathrm{VBPCA}}^{2}$	$3.15\times 10^{-44}$	$\;\mathrm{Reject}\;H_{0}$
	$H_{0}:{\bar{E}}_{\mathrm{MW}-\mathrm{DVBPCA}}^{2}={\bar{E}}_{\mathrm{LSTM}}^{2}$	$8.1\times 10^{-3}$	$\;\mathrm{Reject}\;H_{0}$
	$H_{0}:{\bar{E}}_{\mathrm{MW}-\mathrm{DVBPCA}}^{2}={\bar{E}}_{\mathrm{ESN}}^{2}$	$1.43\times 10^{-18}$	$\;\mathrm{Reject}\;H_{0}$
	$H_{0}:{\bar{E}}_{\mathrm{MW}-\mathrm{DVBPCA}}^{2}={\bar{E}}_{\mathrm{DPLS}}^{2}$	$2.3\times 10^{-3}$	$\;\mathrm{Reject}\;H_{0}$
	$H_{0}:{\bar{E}}_{\mathrm{MW}-\mathrm{DVBPCA}}^{2}={\bar{E}}_{\mathrm{DVBPCA}}^{2}$	$2.58\times 10^{-11}$	$\;\mathrm{Reject}\;H_{0}$
${\mathrm{CO}}_{2}$ concentration	$H_{0}:{\bar{E}}_{\mathrm{MW}-\mathrm{DVBPCA}}^{2}={\bar{E}}_{\mathrm{PLS}}^{2}$	$3.00\times 10^{-35}$	$\;\mathrm{Reject}\;H_{0}$
	$H_{0}:{\bar{E}}_{\mathrm{MW}-\mathrm{DVBPCA}}^{2}={\bar{E}}_{\mathrm{VBPCA}}^{2}$	$1.69\times 10^{-36}$	$\;\mathrm{Reject}\;H_{0}$
	$H_{0}:{\bar{E}}_{\mathrm{MW}-\mathrm{DVBPCA}}^{2}={\bar{E}}_{\mathrm{LSTM}}^{2}$	$8.67\times 10^{-15}$	$\;\mathrm{Reject}\;H_{0}$
	$H_{0}:{\bar{E}}_{\mathrm{MW}-\mathrm{DVBPCA}}^{2}={\bar{E}}_{\mathrm{ESN}}^{2}$	$1.19\times 10^{-5}$	$\;\mathrm{Reject}\;H_{0}$
	$H_{0}:{\bar{E}}_{\mathrm{MW}-\mathrm{DVBPCA}}^{2}={\bar{E}}_{\mathrm{DPLS}}^{2}$	$9.95\times 10^{-4}$	$\;\mathrm{Reject}\;H_{0}$
	$H_{0}:{\bar{E}}_{\mathrm{MW}-\mathrm{DVBPCA}}^{2}={\bar{E}}_{\mathrm{DVBPCA}}^{2}$	$2.78\times 10^{-4}$	$\;\mathrm{Reject}\;H_{0}$
$H_{2}$ concentration	$H_{0}:{\bar{E}}_{\mathrm{MW}-\mathrm{DVBPCA}}^{2}={\bar{E}}_{\mathrm{PLS}}^{2}$	$6.52\times 10^{-74}$	$\;\mathrm{Reject}\;H_{0}$
	$H_{0}:{\bar{E}}_{\mathrm{MW}-\mathrm{DVBPCA}}^{2}={\bar{E}}_{\mathrm{VBPCA}}^{2}$	$4.60\times 10^{-69}$	$\;\mathrm{Reject}\;H_{0}$
	$H_{0}:{\bar{E}}_{\mathrm{MW}-\mathrm{DVBPCA}}^{2}={\bar{E}}_{\mathrm{LSTM}}^{2}$	$3.04\times 10^{-60}$	$\;\mathrm{Reject}\;H_{0}$
	$H_{0}:{\bar{E}}_{\mathrm{MW}-\mathrm{DVBPCA}}^{2}={\bar{E}}_{\mathrm{ESN}}^{2}$	$2.97\times 10^{-29}$	$\;\mathrm{Reject}\;H_{0}$
	$H_{0}:{\bar{E}}_{\mathrm{MW}-\mathrm{DVBPCA}}^{2}={\bar{E}}_{\mathrm{DPLS}}^{2}$	$4.25\times 10^{-30}$	$\;\mathrm{Reject}\;H_{0}$
	$H_{0}:{\bar{E}}_{\mathrm{MW}-\mathrm{DVBPCA}}^{2}={\bar{E}}_{\mathrm{DVBPCA}}^{2}$	$3.19\times 10^{-50}$	$\;\mathrm{Reject}\;H_{0}$

**Table 5:** Comparison of computational efficiency of various models (seconds).

	${\mathrm{CPT}}^{\mathrm{opt}}$	${\mathrm{CPT}}^{\mathrm{online}}$
PLS	3.44	<0.01
VBPCA	11.49	<0.01
LSTM	3893.01	0.04
ESN	264.38	<0.01
DPLS	255.83	<0.01
DVBPCA	210.16	<0.01
MW-DVBPCA	1877.91	0.06

## Data Availability

Data are available for reasonable reasons by contacting the corresponding author.
